# Hypoxia-Induced circRNAs in Human Diseases: From Mechanisms to Potential Applications

**DOI:** 10.3390/cells11091381

**Published:** 2022-04-19

**Authors:** Qi Huang, Juan Yang, Robby Miguel Wen-Jing Goh, Mingliang You, Lingzhi Wang, Zhaowu Ma

**Affiliations:** 1 School of Basic Medicine, Health Science Center, Yangtze University, 1 Nanhuan Road, Jingzhou 434023, China; 201971507@yangtzeu.edu.cn (Q.H.); 202071866@yangtzeu.edu.cn (J.Y.); 2 Watford General Hospital, Vicarage Road Watford Herts, Watford WD18 0HB, UK; r.goh@nhs.net; 3 Key Laboratory of Clinical Cancer Pharmacology and Toxicology Research of Zhejiang Province, Hangzhou Cancer Institute, Hangzhou 310002, China; youml@zjhealth.org; 4 Affiliated Hangzhou Cancer Hospital, School of Medicine, Zhejiang University, Hangzhou 310002, China; 5 Department of Pharmacology, Yong Loo Lin School of Medicine, National University of Singapore, Singapore 117600, Singapore; 6 Cancer Science Institute of Singapore, National University of Singapore, Singapore 117599, Singapore; 7 NUS Center for Cancer Research (N2CR), Yong Loo Lin School of Medicine, National University of Singapore, Singapore 117599, Singapore

**Keywords:** circular RNAs, hypoxic microenvironment, human diseases, clinical implications

## Abstract

Circular RNAs (circRNAs) are a special class of endogenous RNAs characterized by closed loop structures lacking 5′ to 3′ polarity and polyadenylated tails. They are widely present in various organisms and are more stable and conserved than linear RNAs. Accumulating evidence indicates that circRNAs play important roles in physiology-related processes. Under pathological conditions, hypoxia usually worsens disease progression by manipulating the microenvironment for inflammation and invasion through various dysregulated biological molecules. Among them, circRNAs, which are involved in many human diseases, including cancer, are associated with the overexpression of hypoxia-inducible factors. However, the precise mechanisms of hypoxic regulation by circRNAs remain largely unknown. This review summarizes emerging evidence regarding the interplay between circRNAs and hypoxia in the pathophysiological changes of diverse human diseases, including cancer. Next, the impact of hypoxia-induced circRNAs on cancer progression, therapeutic resistance, angiogenesis, and energy metabolism will be discussed. Last, but not least, the potential application of circRNAs in the early detection, prognosis, and treatment of various diseases will be highlighted.

## 1. Introduction

It is currently known that protein-coding genes make up less than 2% of all human genomes, while the majority of nucleotide sequences transcribe noncoding RNAs (ncRNAs) that lack protein-coding functions [1]. Noncoding RNAs include microRNAs (miRNAs), PIWI-interacting RNAs, long non-coding RNA (lncRNA), circular RNAs (circRNAs), and pseudogenes [2]. The concept of a circRNA that lacks 5′ and 3′ polarity and a polyadenylated tail was first proposed in 1976 [3]. Numerous circRNAs have been identified using high-throughput sequencing in eukaryotes, where they have been found to have tissue-specific expression patterns [4]. Studies have demonstrated that circRNAs play diverse roles in the cell cycle, migration, drug resistance, and tumor angiogenesis [5,6,7]. Recently, many circRNAs have also been shown to be involved in various pathophysiological processes and in hypoxia.

Oxygen is essential for normal physiological and metabolic functions in living beings. The energy production machinery and numerous enzyme cofactors/substrates are dependent on oxygen [8]. Hypoxia occurs not only during ischemia, inflammation, and injury but can also manifest in a tumor microenvironment [9]. Cells have developed a complex and necessary adaptive mechanism for survival under hypoxic conditions. Hypoxia-inducible factor (HIF) is the central regulator of oxygen detection and adaptation [10], which regulates the genes associated with cellular oxygen homeostasis, including those involved in angiogenesis, metabolism, and red blood cell production [8] While the regulation of HIF-1-mediated signal transduction and the accompanying transcriptional response have been widely characterized, the various phenotypic changes and downstream events consequent to hypoxia still remain unexplored. Hypoxia is a hallmark of solid tumors, which facilitates the development of aggressive, metastatic, and treatment-resistant tumors by triggering downstream oncogenes and related pathways [11,12]. Emerging evidence from preclinical studies propose strategies that target HIFs as potential therapeutic interventions for many tumors [13]. Several studies have shown that circRNAs can regulate the phenotypes associated with diseases in hypoxic microenvironments. Additionally, circRNAs play complex and heterogeneous roles in various diseases on account of their inherent heterogeneity [14,15,16].

Although recent reviews have focused on the diverse roles of miRNAs and lncRNAs in hypoxic regulation [9,17,18,19,20], the mechanisms and functions of circRNAs on disease progression have not been systematically analyzed. Herein, we summarize the current research on the regulatory roles of circRNAs in hypoxic environments. Furthermore, we also highlight the molecular mechanisms underlying circRNA functions and their potential clinical applications in the diagnosis and treatment of human diseases.

## 2. circRNAs Function as Novel Regulators in Hypoxia

### 2.1. Basic Features and Functions of circRNAs

The first circRNA to be discovered was a viroid linked by host cell enzymes [3,21]. Subsequently, others were detected in eukaryotic cells using electron microscopy. More recently, the importance of circRNAs in terms of their characteristics and functions have come to the forefront [22]. Owing to their tissue- or developmental stage-specific expressions, circRNAs are promising biomarkers for many human diseases. Current studies show that circRNAs can be classified into four types, namely exon-internal circRNA (EIciRNA) [23], exonic circRNAs (ecircRNAs) [24], intronic circRNAs (ciRNAs) [25], and circRNAs produced from tRNAs (tricRNAs) [26].

circRNAs have been found to regulate gene expression and are characterized by several features, including: (i) their highly conserved nature irrespective of the evolutionary distance among species. For instance, approximately 15,000 circRNAs are expressed from mouse and human orthologous loci, representing 40% and 15% of the total circRNAs in mice and humans, respectively; (ii) their high abundance, with expression levels 10 times higher than those of the corresponding linear mRNAs. These molecules are found in fruit flies, mice, plants, archaea, and humans. The balance between circRNA production, nuclear output, and turnover efficiency results in the steady-state abundance of circRNAs [3]; (iii) their tissue- and developmental stage-specific expression profiles [22], (iv) and lastly, their greater stability than linear RNAs, on account of lacking free terminals that confer RNase R resistance and having a covalent closed-loop structure [27].

**Figure 1:**
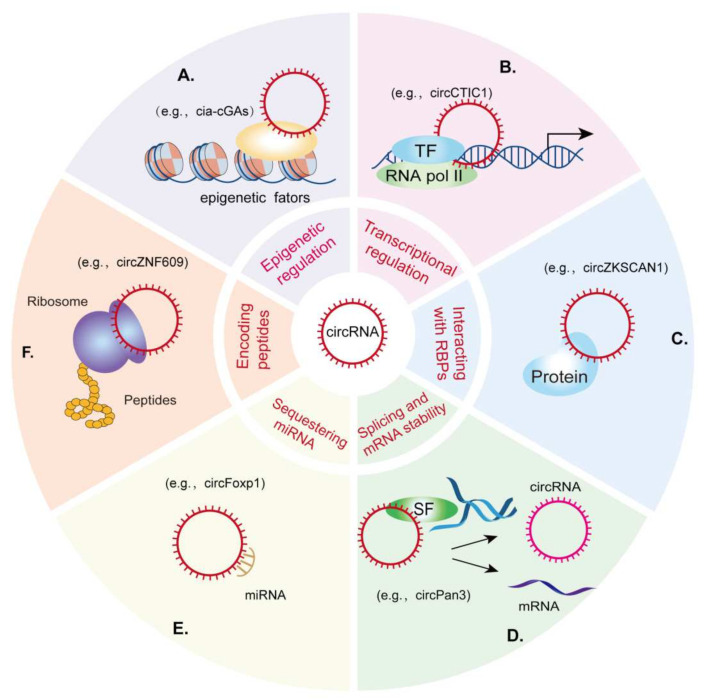
Molecular mechanisms of circRNA functions. circRNAs are involved in (**A**) epigenetic regulation, (**B**) transcriptional regulation, (**C**) protein interactions, (**D**) the regulation of splicing, (**E**) functioning as miRNA sponges, and (**F**) encoding peptides.

Several biological functions of circRNAs have been identified (Figure 1), including: (i) the recruitment of various epigenetic factors to coordinate signal transduction and gene transcription [28]; (ii) interactions with target gene promoters and transcription factors or cofactors to regulate gene transcription [29]; (iii) the regulation of the competitive interactions of mRNAs and pre-mRNAs with splicing factors, transfer RNA binding proteins, or miRNAs [30,31]; (iv) the regulation of a protein or RNA modifications to influence their activation and stability [32,33]; (v) and finally, encoding functional peptides that play key roles in various biological processes [34]. Therefore, circRNA-mediated gene regulation is a complex biological process that participates in a diverse array of diseases, thereby providing avenues for the development of prospective therapeutic interventions.

### 2.2. circRNAs Are New Players for Hypoxic Response

HIF is a transcription factor that senses and adapts to changes in the intracellular oxygen level. It is a heterodimer consisting of an alpha and a beta subunit (the expression of the alpha subunit is oxygen-dependent, whereas the beta subunit is constitutively expressed). Currently, three known alpha subunits (HIF-1α, HIF-2α, and HIF 3α) and a beta subunit (HIF-1β) are known [35,36]. HIFs play relatively more important roles under hypoxic conditions than under normal or high-oxygen conditions [37,38,39,40]. In normoxia, HIF-1α is degraded, and the reports by Ratcliffe and Kaelin showed that prolyl hydroxylases (PHDs) and von Hippel-Lindau (VHL), a tumor suppressor, play important roles in this process. PHD regulates the oxygen-dependent hydroxylation of HIF-1α at two proline residues, namely Pro564 and Pro402. These hydroxylated prolines are recognized by the VHL E3 ubiquitin ligase, and HIF-1α is subsequently ubiquitinated, followed by rapid degradation by the proteasome [37,41]. In hypoxic conditions, PHDs lack access to their co-substrate oxygen and are consequently inhibited. This results in the accumulation of HIF-1α, which subsequently enters the nucleus. In the nucleus, HIF-1α binds to HIF-1β (aryl hydrocarbon receptor nuclear translocator) and activates the associated genes. HIF plays important roles in various physiological and pathological processes, including those associated with immunity, inflammation [41], diabetes [37], atherosclerosis [35], and intestinal disease [38]. To date, a phase III study of the HIF-2α inhibitor MK-6482 is underway on advanced clear cell renal cell carcinoma (NCT04195750), suggesting that targeting HIF-2α could be a promising strategy in a clinical setting. Recently, circRNAs have been established as regulators in hypoxia and have been shown to play multifunctional roles in multiple cellular processes. This review focuses on the emerging roles of circRNAs in the hypoxic microenvironment.

An increasing body of evidence indicates that the crosstalk between hypoxia and circRNAs mediates the diverse processes involved in cellular metabolism, proliferation, and apoptosis. On the one hand, certain circRNAs regulate disease progression by directly mediating the expression of HIFs or affecting downstream pathways [8]. For example, Chi et al. demonstrated that circPIP5K1A activates HIF-1α by adsorbing miR-600, thereby regulating the metastasis and proliferation of non-small cell lung cancer (NSCLC) [42]. Tan et al. showed that the overexpression of circ-EPHB4 resulted in the inhibition of hepatocellular carcinoma (HCC) progression by regulating the HIF-1α expression and the phosphatidylinositol 3 kinase (PI3K)-protein kinase B (AKT) pathways [43]. Furthermore, the results reported by Dang et al. revealed that has-circ-0010729 regulated the apoptosis and proliferation of vascular endothelial cells via the miR-186/HIF-1α axis [15].

On the other hand, hypoxia influences the expression of various circRNAs, thus affecting the development and progression of various diseases. For instance, a hypoxia-responsive circRNA, circDENND2A, facilitates glioma aggressiveness by sponging miR-625-5p [14]. Similarly, hypoxia induces has-circRNA-403658 expression in bladder cancer and circDENND4C expression in breast cancer [44,45]. These hypoxia-regulated circRNAs influence the progression of cancer via various mechanisms. Therefore, the recent findings on circRNAs will improve our understanding of hypoxic regulation and enable the identification of novel therapeutic targets for various diseases.

**Figure 2:**
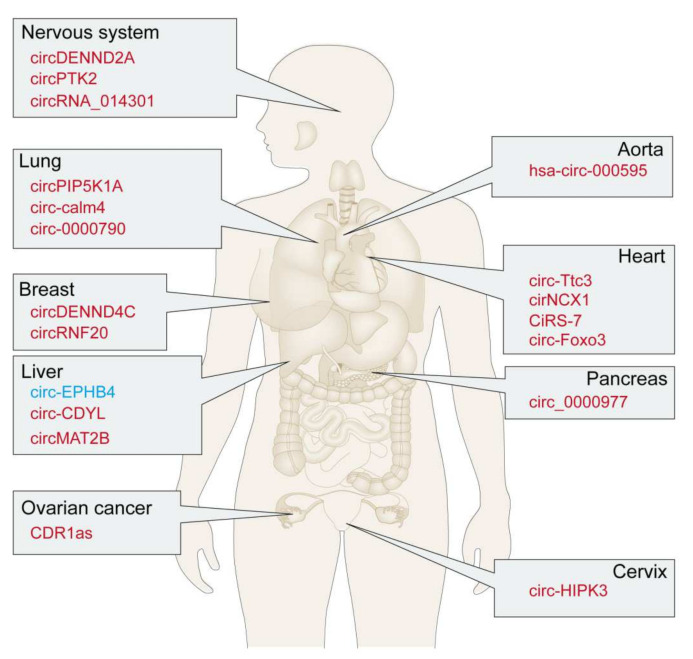
Emerging roles of circRNAs in hypoxic regulation. circRNAs perform diverse roles in hypoxic regulation in various tissues, including the lungs, liver, and breasts. Upregulated circRNAs are shown in red, whereas downregulated circRNAs are in blue.

We summarized the current information available about the contribution of hypoxia-related circRNAs in various diseases (Table 1 and Figure 2). A comprehensive understanding of this will potentially aid in the development of novel and promising therapeutic options for clinical applications.

### 2.3. Emerging Roles of circRNAs in Pathological Responses to Hypoxia

Several studies have conclusively proven that circRNAs are related to the pathogenesis of various diseases affecting the cardiovascular, pulmonary, nervous, and gynecological systems (Figure 2). Under hypoxia, these molecules play fundamental roles in disease progression via unique mechanisms [76,77].

The physiological and pathological processes of many cardiac diseases are affected by hypoxia. Hypoxia is the driving force that is responsible for the characteristic metabolic switch from the oxidation of fatty acids in a healthy heart to the utilization of glucose in a failing myocardium. It also promotes the reactivation of fetal gene programs, thereby inducing the cardiac hypertrophy response, changing the composition of the extracellular matrix, and affecting mitochondrial biogenesis, as well as myocardial contractility. Hypoxia-related circRNAs may add to the complexities involved in the regulation of hypoxia-mediated effects, and unraveling the roles played by these circRNAs may provide new directions for the treatment of cardiovascular diseases [78]. The first study to reveal the biological function of a circRNA in the heart was published in 2016 [79]. Subsequently, Li et al. discovered that circNCX1 was related to cardiomyocyte apoptosis induced by oxidative stress. This effect is mediated by the sponging of miR-133a-3p by circNCX1, which decreases the inhibitory activity of the proapoptotic gene encoding the cell death-inducing protein and promotes myocardial ischemia–reperfusion (I/R) injury and apoptosis [68]. Cdr1as (or CiRS-7) has been shown to be significantly upregulated in cardiomyocytes undergoing either hypoxia treatment or myocardial infarction, where it regulates the expression of SP1 and PARP by sponging miR-7a to promote cardiomyocyte apoptosis [69]. Similarly, circ-Ttc3 is upregulated in hypoxic-ischemic cardiomyocytes. A knockdown of circ-Ttc3 promotes hypoxia-induced apoptosis and ATP consumption. These findings indicate that the circ-Ttc3/miR-15b/Arl2 axis can protect the heart during myocardial infarction [70]. Du et al. demonstrated the high-level expression of circ-Foxo3 in the heart, which is related to cellular senescence, and the knockdown of circ-Foxo3 inhibited senescence in fibroblasts. Mechanistically, circFoxo3 harbors binding sites for various proteins and interacts with E2F1 (E2F transcription factor 1), FAK (focal adhesion kinase 1), anti-stress transcription factor ID1 (DNA-binding protein inhibitor ID-1), and HIF-1α to promote cell senescence. Additionally, the ectopic expression of circ-Foxo3 induces senescence [16]. Another study demonstrated that hsa-circ-000595 expression is significantly upregulated in hypoxic aortic smooth muscle cells and promotes apoptosis in the same by adsorbing miR-19a [71]. Thus, circRNAs exert negative or protective influences on the progression of cardiac remodeling by various mechanisms. An in-depth understanding of the circRNA-induced mechanisms may, therefore, provide novel ideas for the treatment of heart diseases.

Long-term hypoxia in the alveoli, as seen in chronic lung disease, induces pulmonary vascular remodeling and results in increased pulmonary artery pressure and right ventricular hypertrophy. Various HIF-1 target genes have been confirmed to play critical roles in the response of pulmonary artery smooth muscle cells to hypoxia [80]. Certain circRNAs have a broad repertoire of functions in lung diseases that ultimately reshape hypoxic microenvironments. For instance, circ-calm4 is abnormally expressed in hypoxic lung tissue and regulates the proliferation of pulmonary artery smooth muscle cells (PASMC). The circ-calm4/miR-337-3p/Myo10 signal transduction axis modulates the proliferation of PASMC at the molecular level, thus establishing potential targets for the early diagnosis and treatment of pulmonary hypertension [72]. Similarly, mmu-circ-0000790 is upregulated in PASMCs during HPH, which regulates FOXC1 expression by sponging miR-374c to subsequently promote hypoxia-induced PASMC growth and suppress apoptosis [73]. Another study assessed the circRNA expression profile (circRNA_004592 and circRNA_018351) in HPH, albeit without a detailed functional analysis [81].

The high stability of circRNAs is responsible for their abundant expression in the central nervous system. Certain circRNAs have been implicated in inflammatory diseases or immune disorders of the nervous system, especially in hypoxia-induced neurological diseases [3,75,81]. For instance, circPTK2 plays a key role in oxygen-glucose deprivation-activated microglia-induced hippocampal neuronal apoptosis. Mechanistically, circPTK2 inhibits SOCS-1 expression by competitively binding to miR-29b, consequently upregulating JAK2/STAT3 signaling and suppressing IL-1β expression, thereby promoting neuronal apoptosis [74]. Similarly, hypoxia-related circRNAs are differentially expressed in heat adaptation, hypoxic-ischemic brain damage, and moyamoya disease [82,83,84,85].

Preliminary research on circRNAs in the context of other diseases is currently underway. circ-Ttc3 has been shown to regulate the nuclear factor-kappa B and PI3K-AKT pathways by competitively binding to miR-449a and reducing hypoxic damage in pressure ulcers [75]. circRNAs may be directly involved in hypoxic microenvironments or the HIF-1 pathway in multiple uterine disorders. For instance, hsa-circ-0015382 and hg38-circ-0014736 have been confirmed to be upregulated, whereas hsa-circ-0007121 is downregulated in preeclampsia [86]. Similarly, circ-103470 and circ-101102 may regulate the epithelial–mesenchymal transition (EMT) during endometriosis by adsorbing miR-141-5p to influence HIF-1 signal transduction [87]. Furthermore, experimental studies have demonstrated that circRNA-102399/miR-302a-3p/HIF1A may be a key target for the treatment of intervertebral disc degeneration [88]. The dysregulation of circRNAs may, therefore, play a significant role in the pathogenesis of various diseases induced by hypoxia. In view of the fact that the current studies on hypoxia-related circRNAs are still in their infancy, targeting these small molecules for clinical applications in the present scenario is difficult. The establishment of a comprehensive understanding of hypoxia-related circRNAs in the context of the complex pathogenesis associated with various diseases will aid in their therapeutic applications.

## 3. Functions of circRNAs in Hypoxic Microenvironments

**Figure 3:**
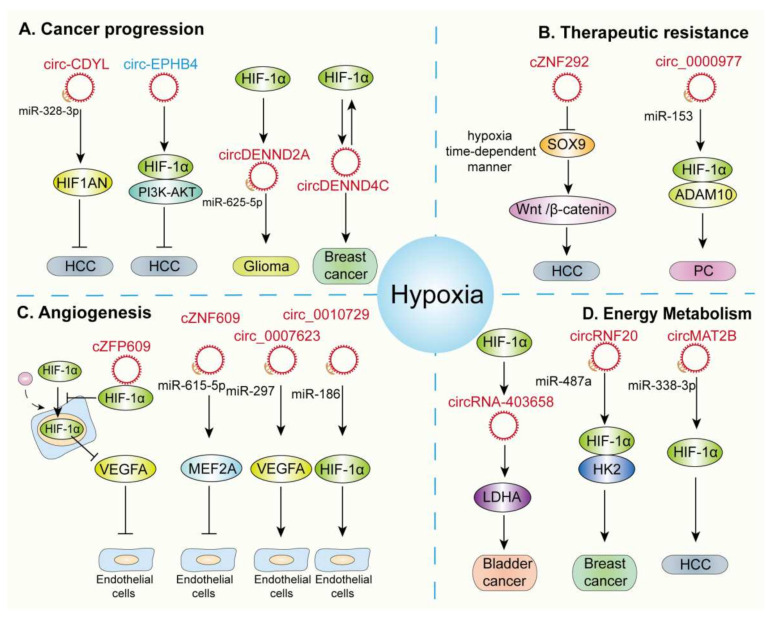
Functions of circRNAs in hypoxic microenvironments. (**A**) circRNAs mediate cancer progression under hypoxia. (**B**) circRNAs regulate therapeutic resistance. (**C**) Hypoxia-induced circRNAs control angiogenesis. (**D**) Hypoxia-induced circRNAs influence energy metabolism. The upregulated circRNAs are in red, whereas the downregulated circRNAs are in blue.

Multiple studies have indicated that circRNAs play pivotal roles in the establishment of hypoxic microenvironments. These molecules regulate physiological and pathological pathways, including cancer progression and metabolism. The following sections summarize the role of hypoxic circRNAs in cancer progression, therapeutic resistance, metabolism, and angiogenesis (Figure 3).

### 3.1. Hypoxic circRNAs in Cancer Progression

Recent research on RNA molecules has revealed that the dynamics of cancer-related gene expression are highly complicated [89]. CircRNAs are involved in the regulation of cancer progression, metabolism, and immune escape [6,7,89]. It is well-known that hypoxic microenvironments are related to tumor progression, metabolism, drug resistance, and angiogenesis [13,90] and play a critical role in the function of circRNAs in tumors. Although preliminary studies regarding the universality of the interactions between circRNAs and hypoxia/HIF are underway, further in-depth studies are essential to identify novel cancer biomarkers and therapeutic targets.

Recent studies have indicated that circRNAs regulate cancer progression by regulating various hypoxia-related molecules (Figure 3A). For instance, Wei et al. reported that circ-CDYL is upregulated in HCC and is a competing endogenous RNA for miR-328-3p and miR-892a via its interactions with mRNA encoding hypoxia-inducible factor 1-α inhibitor (HIF1AN) and hepatoma-derived growth factor (HDGF). The subsequent activation of the PI3K-AKT serine/threonine kinase mTOR kinase complex 1/β-catenin and NOTCH2 pathways promotes the expression of the effector proteins, baculoviral IAP repeat containing 5 (BIRC5 or SURVIVIN), and MYC proto-oncogene. Therefore, the overexpression of circ-CDYL results in the self-renewal and malignant proliferation of liver cells [46]. Another study demonstrated that the overexpression of cytoplasmic circPIP5K1A promotes the proliferation and metastasis of NSCLC, since it adsorbs miR-600, which interacts with the 3′-untranslated region (UTR) of HIF-1α [42]. A similar study showed that circ-HIPK3 is upregulated in CC cells, where it functions as a miR-338-3p sponge to subsequently upregulate the expression of HIF-1α, thereby contributing to the progression and metastasis of CC [47]. CircC6orf132 promotes gastric cancer proliferation, migration, invasion, and glycolysis under hypoxic conditions. Mechanistically, CircC6orf132 adsorbs miR-873-5p and elevates the expression of PRKAA1, a protein kinase AMP-activated alpha 1 catalytic subunit [48]. circSETDB1, a hypoxic tumor-derived exosomal circRNA, is upregulated in lung adenocarcinoma (LUAD), which is associated with the LUAD stage in serum exosome patients. circSETDB1 promotes LUAD development and EMT via the miR-7/Sp1 axis [49]. Feng et al. reported that hsa-circ-0000211 is upregulated in LUAD cells and promotes the migration of LUAD via the miR-622/HIF1-α axis [50]. Additionally, Su et al. confirmed that hypoxia promotes the expression of circDENND2A in gliomas. Functional assays indicate that circDENND2A promotes the invasion and migration of gliomas by sponging miR-625-5p [14]. Furthermore, Liang et al. demonstrated the elevation of the circDENND4C levels in breast cancer cells. In vitro assays have established that silencing circDENND4C inhibits the proliferation of breast cancer cells in hypoxic microenvironments [45]. circZFR is another circRNA that promotes the malignant progression of breast cancer. The silencing of circZFR inhibits BC cell viability, colony formation, migration, invasion, and glycolysis via the miR-578/HIF1A axis [51]. A recent study revealed that circHIF1A (hsa_circ_0004623) promoted cancer cell proliferation and metastasis in triple-negative breast cancer (TNBC). Mechanistically, circHIF1A regulates the expression and translocation of NFIB through post-transcriptional and post-translational modifications, resulting in activation of the AKT/STAT3 signaling pathway and the repression of P21. The RNA-binding protein FUS regulates the biosynthesis of circHIF1A by interacting with flanking introns, and FUS is transcriptionally regulated by NFIB, forming a circHIF1A/NFIB/FUS positive feedback loop [52].

Contrary to the above-mentioned circRNAs that promote the occurrence and development of HCC, circ-EPHB4 significantly inhibits the development of HCC. Functional assays have shown that the circ-EPHB4 levels decrease in HCC, which inhibits the occurrence, development, and metastasis of HCC by modulating the PI3K-AKT pathway and HIF-1α [43]. Similarly, CDR1as functions as a tumor suppressor and is poorly expressed in ovarian cancer. CDR1as promotes the expression of HIF1AN by sequestering miR-135b-5p, thereby inhibiting the proliferation of ovarian cancer cells [53]. Therefore, these studies indicate that circRNAs may positively or negatively affect tumor progression in hypoxic microenvironments.

### 3.2. Hypoxic circRNAs Regulate Therapeutic Resistance

Currently, cancer treatment is synonymous with chemotherapy, radiotherapy, and immunotherapy. However, radiotherapy and chemotherapy have many limitations, including disease relapse and metastasis due to the development of therapeutic resistance [91]. These effects in patients undergoing radiotherapy and chemotherapy may be related to primary, secondary, or acquired resistance [92]. Extrinsic factors within the tumor microenvironment that promote resistance to chemoradiotherapy include hypoxia, the extracellular matrix, and angiogenesis. circRNAs may also potentially play a role in these processes [22]. Several studies have demonstrated an association between circRNAs in hypoxic tumor microenvironments and resistance to radiotherapy and chemotherapy, which culminates in adverse clinical outcomes (Figure 3B).

A recent study reported that cZNF292 played a promotive role in HCC in a time-dependent manner independent of HIF-1α. The knockdown of cZNF292 resulted in an increase in the nuclear translocation of SRY (sex-determining region Y)-box 9 (SOX9) and simultaneously downregulated the Wnt/β-catenin pathway, thereby inhibiting radioresistance and decreasing the proliferation of hypoxic hepatocytes [54]. Joseph et al. indicated that cirRNA CCDC66 is upregulated in LADC on account of the negative regulation of nAchR7α and positive regulation of c-Met and FAK. The immediate responses to hypoxia include the upregulation of phosphorylated c-Met, EMT, and SAE2, which results in a greater resistance and EMT of LADC [55]. Another study on cancer drug resistance showed that the circELP3 levels were elevated in bladder cancer in hypoxic microenvironments in correlation with the grade and metastatic stage of the tumor. Bladder cancer with a low expression of circELP3 had a poor self-renewal ability, and silencing of the same increased the apoptosis of bladder cancer cells while decreasing the resistance to cisplatin [56]. Hypoxic microenvironments also play a causal role in radioresistance and the metastasis of pancreatic cancer (PC). For instance, circZNF91 experiments that transferred a hypoxia-induced exosomal circRNA into normoxic PC cells to promote the expression of the deacetylase SIRT1 resulted in the subsequent SIRT1-mediated facilitation of glycolysis and gemcitabine chemoresistance in the recipient PC cells via enhancement of the deacetylation-dependent stability of HIF-1α [57]. Additionally, hypoxia upregulates circ-0000977 in PC, thereby aiding the evasion of immune responses. Overexpressed circ-0000977 has been shown to function as a miR-153 sponge and upregulates its downstream targets, namely ADAM10 and HI1FA. This results in the suppression of the cytotoxic effects of natural killer cells in hypoxic microenvironments [58].

### 3.3. Hypoxic circRNAs Regulate Angiogenesis

Blood vessels provide nourishment and oxygen to tissues in the human body and are lined by endothelial cells (EC). While those in a healthy human body are stable, the formation of new blood vessels is aggressively promoted to deliver nutrients and oxygen to hypoxic tissues under pathological conditions [93]. Angiogenesis is a complex multi-step process that is stimulated by various proangiogenic factors (such as vascular endothelial growth factor, VEGF), where the original dynamic balance of the vascular network is perturbed, the capillary basement membrane is degraded, and the ECs migrate and proliferate, resulting in the formation of new primary capillary networks [94,95]. Angiogenic factors, including members of the VEGF family, such as vascular endothelial growth factor A (VEGFA), and the vascular endothelial growth factor receptor (VEGFR) family, promote EC migration and proliferation. Several studies have demonstrated that hypoxia-responsive circRNAs affect the proliferation or apoptosis of ECs by regulating the downstream targets [59,61]. Here, we summarize the available research on the regulation of ECs by hypoxia-related circRNAs (Figure 3C).

VEGFA, a key regulator of angiogenesis, is regulated by certain oncogenes and transcription factors (HIF-1) during hypoxic stress [96]. The activation of VEGFR-1 and VEGFR-2 mediates the proangiogenic activity of VEGFA [97]. Studies have demonstrated that circRNAs can induce phenotypic changes as a part of hypoxic regulation. For instance, Boeckel et al. reported that cZNF292 promoted angiogenesis in hypoxia in vitro. The overexpression of cZNF292 resulted in increased globular sprouting and the tube formation of ECs [59]. Furthermore, circ-Erbin facilitates angiogenesis via the miR-125a-5p-5p/miR-138-5p/4EBP-1 axis and contributes to HIF-1α activation in CRC [60]. circRNAs can positively or negatively modulate the proliferation of ECs in hypoxic microenvironments by adsorbing miRNAs or by directly acting on VEGFA. For instance, the overexpression of cZBTB44 promotes EC viability, proliferation, migration, and tube formation under hypoxic stress in vitro. Mechanistically, cZBTB44 increases the expression of VEGFA and vascular cell adhesion molecule 1 (VCAM1) by competitively binding to miR-578 in order to regulate EC functions [61]. Similarly, hsa-circ-0007623 expression is elevated in human umbilical vein endothelial cells (HUVECs) under hypoxia stress, which facilitates EC proliferation, migration, and angiogenesis. Further, the sponging of miR-297 by hsa-circ-0007623 promotes VEGFA expression. The hsa-circ-0007623/miR-297/VEGFA axis aids in the repair of the heart after acute myocardial ischemia and plays a protective role [62]. On the contrary, cZFP609 inhibits endothelial angiogenic function in response to hypoxia. Mechanistically, exosomal cZFP609 is delivered to ECs from the VSMCs, thereby suppressing angiogenesis post-ischemia via the inhibition of HIF-1α activation [63].

circRNAs are also known to exert their modulatory effects on EC via other molecules (MEF2A, IGF-1, and HIF-1α). cZNF609 is upregulated in low-oxygen and high-glucose environments and enhances the expression of its downstream target MEF2A by competitively binding to miR-615-5p. The cZNF609/miR-615-5p/MEF2A axis has been confirmed to inhibit the tube formation, proliferation, and migration of ECs [64]. Dang et al. found that hsa-circ-0010729 interacts with miR-186/HIF-1α to promote the proliferation and migration of HUVECs [15]. Furthermore, the expression of circHIPK3 is elevated under oxidative conditions in vitro. circHIPK3 adsorbs miR-29a to promote the expression of IGF-1, thereby reducing CMVEC dysfunction induced by oxidative stress [65].

### 3.4. Hypoxic circRNAs Influence Energy Metabolism

The energy metabolism of tumor cells differs from that of normal cells in that they preferentially utilize glucose. This is known as the Warburg effect, which results in increased glycolysis and lactic acid production irrespective of the oxygen availability [98]. The Warburg effect is considered one of the emerging signs of cancer, as it endows tumor cells with specific metabolic characteristics while rendering them resistant to apoptosis and rapid growth [99]. Hypoxia signals induce tumor cells to reprogram gene expression and metabolic activities via the transcriptional regulation of HIFs. Reprogramming of the glucose flux is critical in establishing the tumor microenvironment, since it results in the elevation of the HIF-1α levels in response to hypoxia in rapidly growing cancer cells [10].

circRNAs influence the energy metabolism by regulating a series of signal transduction pathways in the pathological responses to hypoxia (Figure 3D). For instance, circMAT2B was reported to be upregulated in HCC, where it sequesters miR-338-3p and upregulates the downstream gene, *PKM2*. *PKM2*, in turn, encodes a key enzyme involved in glycolysis and HCC progression [66]. Another study confirmed that circRNF20 was markedly upregulated in breast cancer and promoted the Warburg effect and cellular proliferation. Mechanistically, circRNF20 competitively binds to miR-487a and elevates HIF-1α expression and promotes the transcription of hexokinase II (HK2), thereby regulating the Warburg effect and proliferation of breast cancer cells [67]. CircDENND4C is another hypoxia-related circRNA that regulates breast cancer glycolysis. Functional assays indicate that circDENND4C is upregulated in breast cancer. Mechanistically, circDENND4C promotes glycolysis in, as well as the invasion and migration of, breast cancer in hypoxic microenvironments by competitively binding to miR-200b and miR-200c [100].

### 3.5. Other Regulations by Hypoxic circRNAs

Preliminary investigations have highlighted the role of circRNAs in hypoxic microenvironments of tumors but have not yet elucidated the detailed mechanisms of their actions. For example, 65 circRNAs were identified to be differentially expressed in hypoxic lung cancer cells. Among them, circFAM120A (hsa-circ-0008193) is downregulated and may be involved in the occurrence of lung cancer [101]. A similar study indicated that 12 circRNAs may be involved in the development of osteosarcoma. A functional analysis revealed that hsa-circRNA-103801 is involved in multiple cancer pathways, including the VEGF, angiogenesis, and HIF-1 pathways [102]. Additionally, using an analysis pipeline, Di Liddo et al. found that the expression of 64 circRNAs are markedly altered upon exposure to hypoxic stress in the tumor microenvironment [103]. Although research on the mechanisms of circRNA actions in cancer cell lines in hypoxic microenvironments is limited, hypoxia-related circRNAs have been confirmed to contribute to the complex pathogenesis of tumors. Future research is essential to reveal the mechanistic details of hypoxia-related circRNA functions in various diseases, which may potentially be exploited as biomarkers or therapeutic targets in clinical applications.

## 4. Potential Clinical Applications of circRNAs in Human Diseases

The current methods used for the early detection of cancer, including those that utilize levels of blood biomarkers, carcinoembryonic antigen, and prostate-specific antigen, lack specificity and sensitivity. This severely limits the early diagnosis and treatment of cancer patients. Recently, the detection of circRNAs in tumor biopsies has generated considerable interest [104]. With the widespread use of high-throughput RNA sequencing (RNA-seq), many circRNAs have been postulated to be potential diagnostic biomarkers [22,105]. The characteristics and expression patterns of circRNAs (conservation, specificity, versatility, and stability) make them ideal biomarker candidates [22]. Additionally, circRNAs are found abundantly in saliva and blood samples, which renders sampling and detection easier. Consequently, utilizing circRNAs as disease biomarkers in terms of the early diagnosis, treatment selection, and prediction of recurrence has gradually become a research hotspot [106]. Hypoxia-related circRNAs can be exploited not only as biomarkers but also as potential therapeutic targets. For instance, the combination of circ-CDYL with HIF1AN and HDGF has been shown to be an effective biomarker for HCC, with odds ratios of 1.09 (95% confidence interval (CI), 1.02−1.17) and 124.58 (95% CI, 13.26−1170.56), respectively [46]. Recent studies have indicated that circ-calm4 and mmu-circ-0000790 may be utilized as promising biomarkers for HPH [72,73].

As circRNAs promote or suppress disease progression, strategies that involve the specific targeting of circRNAs may be promising clinical therapeutic options. Currently, RNA-based therapies primarily utilize RNA interference (RNAi) and antisense oligonucleotides, which are designed to target specific regions and diverse RNAs. Gene silencing or overexpression approaches may be used to target circRNAs in preclinical research. Specific shRNAs or siRNAs have been used to target the post-splice junction or the intronic sequences of intron-circularized circRNAs. This process involves the meticulous designing of complementarily paired siRNAs to knock down circRNA expressions [107,108]. For suppressive circRNAs, overexpression vectors that foster back-splicings consist of flanking introns with reverse complementary sequences and circRNA-forming exons [109]. This provides a new repertoire of candidates for RNA-founded therapeutics that target specific circRNAs. Therefore, targeting circRNAs in hypoxic microenvironments may broaden therapeutic strategies for many complex and heterogeneous diseases. For instance, circPIP5K1A and circDENND4C may be promising therapeutic targets for NSCLC and breast cancer, respectively, in hypoxia [42,100]. Additionally, cZBTB44 may be a promising therapeutic target for vascular injury and neovascularization-related diseases induced by hypoxic stress [61]. Therefore, circRNAs are promising therapeutic targets that can be used to promote the development of alternate treatment strategies for multiple disorders.

Although the research on hypoxia-related circRNAs is still in its infancy, it is well-established that hypoxia-mediated circRNAs possess numerous advantages. A combination of circRNAs and traditional biomarkers may result in a higher diagnostic or prognostic accuracy than that of a single traditional biomarker [104,110]. Currently, clinical studies on circRNAs are scarce, and the clinical application of circRNAs faces many challenges. The development of new and improved experimental methods will aid in overcoming these challenges.

## 5. Concluding Remarks and Perspectives

This review emphasizes the roles of circRNAs in hypoxia-induced regulation and its impact on disease progression, as well as the diagnostic and therapeutic potentials in the treatment of various diseases. circRNAs orchestrate disease progression via distinct molecular mechanisms involving various HIFs. Currently, circRNAs with diverse functions have been identified in multiple diseases, and thus, they might be utilized as promising diagnostic and prognostic biomarkers. Although the research on circRNAs in cancer has progressed rapidly, the functions of circRNAs in hypoxia-induced cancer proliferation and metastasis are just beginning to be understood. Extensive studies are urgently required to discover novel cancer treatment strategies through targeting hypoxia-related circRNAs with advanced RNA technologies.

Despite the recent advancements in research on circRNAs in hypoxic microenvironments, certain challenges and limitations are yet to be overcome. Some of the unanswered questions in the field include: (i) What is the precise regulatory mechanism involved in cyclization under hypoxic conditions? (ii) How do we identify the crucial factors that affect the dysregulation of circRNA expressions under hypoxic conditions? (iii) What contributes to the large differences in hypoxic responses between animal models and cell lines of the same cancer? In order to address the abovementioned questions, further research in different models is needed to elucidate the detailed mechanisms of action of circRNAs in hypoxic microenvironments. Research on the biological functions of hypoxia-related circRNAs may provide new biomarkers or targets for various diseases, especially cancer, which will be a major advancement in oncomedicine.

Most of the currently published research findings are focused on the pathogenic mechanisms of circRNAs in hypoxic microenvironments, including those involved in the proliferation of various cell types, tumor cell invasion, immune surveillance, chemical and radiation resistance, and production of vascular ECs. Certain circRNAs are pathogenic in hypoxic microenvironments; other circRNAs may beneficially affect disease processes—for instance, the protection afforded to the heart from myocardial infarction by these molecules [62]. Different circRNAs play multiple and even opposing roles in regulating disease progression via multiple mechanisms, which highlights the heterogeneity of circRNAs. Further studies should include the development of new technologies to aid basic and preclinical investigations to determine the important signaling pathways involved in the hypoxia-mediated regulation of circRNAs. Stringent and accredited standards are also essential for the successful development of hypoxia-related models and research tools to aid in the accurate and in-depth studies of circRNAs in hypoxic microenvironments. It is noteworthy that drugs (e.g., Acriflavine, Gliotoxin, and Vadadustat) that regulate hypoxia-related signaling pathways are currently available [90,111], and the combination of specific circRNAs with these drugs may improve patient outcomes. Together, circRNAs are expected to play crucial roles in the early detection and treatment of various diseases in the future.

## Figures and Tables

**Table 1:** Summary of the circRNAs related to the hypoxic microenvironment.

CircRNA	Interacting Partner	Targets/Pathways	Functions	Diseases/Cells/Organs	Reference
Cancer progression					
Circ-CDYL	miR-328-3p	HIF1AN	Promotes stem-like characteristics and tumor growth in vitro and in vivo	HCC	[46]
CircPIP5K1A	miR-600	HIF-1α	Promotes non-small cell lung cancer (NSCLC) proliferation and metastasis in vitro and in vivo	NSCLC	[42]
Circ-HIPK3	miR-338-3p	HIF-1α	Promotes EMT of cervical cancer (CC) in vitro	CC	[47]
CircC6orf132	miR-873-5p	Protein kinase AMP-activated alpha 1 catalytic subunit (PRKAA1)	Promotes gastric cancer proliferation, migration, invasion and glycolysis under hypoxic conditions in vitro and in vivo	Gastric cancer	[48]
CircSETDB1	miR-7	Specificity protein 1 (Sp1)	Promotes invasive growth and EMT in vitro and in vivo	Lung adenocarcinoma (LUAD)	[49]
hsa-circ-0000211	miR-622	HIF-1α	Promotes lung adenocarcinoma migration and invasion in vitro	LUAD	[50]
CircDENND2A	miR-625-5p		Promotes glioma aggressiveness in vitro	Glioma	[14]
CircDENND4C			Promotes the proliferation of breast cancer cells under hypoxia in vitro	Breast cancer	[45]
CircZFR	miR-578	HIF1A	Promotes breast cancer progression in vitro and in vivo	Breast cancer	[51]
circHIF1A		NFIB and FUS	Promotes TNBC growth and metastasis in vitro and in vivo	Breast cancer	[52]
Circ-EPHB4		HIF-1α and PI3K-AKT pathways	Inhibits tumorigenesis, tumor development, and metastasis in vitro and in vivo	HCC	[43]
CDR1as	miR-135b-5p	HIF1AN	Suppresses ovarian cancer progression in vitro	Ovarian cancer	[53]
Therapeutic resistance					
cZNF292		Wnt/β-catenin pathway	Promotes hypoxic human hepatoma SMMC7721 cell proliferation, vasculogenic mimicry, and radioresistance in vitro and in vivo	Hepatoma	[54]
CirRNA CCDC66			Increases EMT and drug resistance of LADC cells in vitro	LUAD	[55]
CircELP3			Contributes to bladder cancer progression and cisplatin resistance in vitro and in vivo	Bladder cancer	[56]
CircZNF91		Sirtuin1 (SIRT1) and HIF-1α	Facilitates glycolysis and gemcitabine chemoresistance of recipient PC cells in vitro and in vivo	Pancreatic cancer	[57]
Circ_0000977	miR-153	HIF1 and ADAM10	Modulates HIF1A-mediated immune escape of PC cells in vitro	Pancreatic cancer	[58]
Angiogenesis					
cZNF292			Induces tube formation and spheroid sprouting of endothelial cells in vitro	Endothelial cells	[59]
Circ-Erbin	miR-125a-5p and miR-138-5p	4E binding protein 1(4EBP-1)	Facilitates the proliferation, migration, and metastasis of colorectal cancer (CRC) in vitro and in vivo	CRC	[60]
cZBTB44	miR-578	VEGFA/VCAM1	Induces endothelial cell viability, proliferation, migration, and tube formation in vitro and in vivo	Choroidal neovascularization (CNV)	[61]
hsa_circ_0007623	miR-297	VEGFA	Promotes cardiac repair after acute myocardial ischemia and protects cardiac function in vitro and in vivo	Heart	[62]
cZFP609	HIF-1α	VEGFA	Inhibits VEGFA expression and endothelial angiogenic functions in vitro and in vivo	Vascular smooth muscle cells (VSMCs)	[63]
cZNF609	miR-615-5p	MEF2A	Decreases endothelial cell migration and tube formation in vitro and in vivo	Vascular dysfunction	[64]
hsa_circ_0010729	miR-186	HIF-1α	Regulates vascular endothelial cell proliferation and apoptosis in vitro	Human umbilical vein endothelial cells (HUVECs)	[15]
CircHIPK3	miR-29a	IGF-1	Decrease in oxidative stress-induced CMVECs dysfunction in vitro and in vivo	Cardiac microvascular endothelial cells (CMVECs)	[65]
Energy metabolism					
CircMAT2B	MiR-338-3p	PKM2	Promotes HCC progression by enhanced glycolysis in vitro and in vivo	HCC	[66]
circRNF20	miR-487a	HIF-1α/HK2	Promotes the proliferation and aerobic glycolysis in vitro and in vivo	Breast cancer	[67]
Other regulation					
CircNCX1	miR-133a-3p	Cell death-inducing protein (CDIP1)	Promotes cardiomyocyte apoptosis in vitro and in vivo	Cardiomyocyte apoptosis	[68]
Cdr1as	miR-7a	PARP and SP1	Increases the cardiac infarct size in vitro *and* in vivo	Myocardial infarction (MI)	[69]
Circ-Ttc3	miR-15b	Arl2	In cardiomyocytes counteracted hypoxia-induced ATP depletion and apoptotic death in vitro and in vivo	MI	[70]
Circ-Foxo3	ID-1, E2F1, FAK, and HIF1α		Promotes cardiac senescence in vitro and in vivo	Heart	[16]
hsa-circ-000595	miR-19a		Increases the apoptotic rate of human aortic smooth muscle cells in vitro	Aortic smooth muscle cells	[71]
Circ-calm4	miR-337-3p	Myo10 (myosin 10)	Promotes pulmonary artery smooth muscle proliferation in vitro and in vivo	Pulmonary hypertension	[72]
mmu_circ_0000790	miR-374c	Forkhead transcription factor 1 (FOXC1)	Induces proliferation and inhibits apoptosis of hypoxic PASMCs in vitro and in vivo	Hypoxic pulmonary hypertension (HPH)	[73]
CircPTK2	miR-29b	SOCS-1-JAK2/STAT3-IL-1β	Regulates oxygen-glucose deprivation-activated microglia-induced hippocampal neuronal apoptosis in vitro and in vivo	Microglia	[74]
Circ-Ttc3	miR-449a	NF-κB and PI3K/AKT pathways	Alleviates hypoxic injury in vitro	HaCaT cells	[75]

## Data Availability

Not applicable.
